# On Hallucinations in Their Relation to Medical Jurisprudence

**Published:** 1862-01

**Authors:** A. Brierre de Boismont


### Art. IV.—ON HALLUCINATIONS IN THEIR RELATION TO MEDICAL JURISPRUDENCE.

By A. Brierre de Boismont.

(*Continued from* No. IV., p. 668.)

Not only are figures, objects of sight, metamorphosed to the subjects of hallucination, but words, and auditions, in the absence of sound, submit to the same transformations, and enter into the system of morbid interpretation peculiar to the insane. These strange modifications constitute for the patient a fantastical world in which he lives exclusively, to which all his impressions are related, whilst the actual world is to him a mere secondary matter. One of the most curious facts of this nature is the case of one of our boarders, who pretended that every one treated her as an idiot or a fool; if any one laughed or spat, it was to scorn or insult her. The cries of the street, the barking of dogs, the neighing of horses, the cracking of whips, the breaking of anything, were so many proofs of malevolence against her; her words, her thoughts, were instantly incriminated. If she appeared at a window, people withdrew. As to everything else, her reason was perfect. We were acquainted with another lady who imagined that coachmen, horses, carriages stopped or made burlesque attitudes at her. The belief of the hallucinated with regard to this class of subjects is uncontrolled either by reason or passion, because there is no counterpoise which can weigh down the balance. Thus mastered by his delirious convictions he may commit any crime. Of him, above all, it may be said, Woe to him who crossing his path he shall believe to be his enemy!

The observations respecting this class are so important towards the demonstration of these propositions, that we shall relate a few of them.

Amongst the facts which attest the terrible consequences of these personal changes we would mention the following:—

M. H. C., after a mental malady from which he had not completely recovered, returned to his family. The next day, he killed his wife and his sister-in-law, whom he took for devils.

He was sent to Charenton, afterwards, in 1825, to the private establishment of M. Marcel Saint-Colombe, where I was physician, and where he lived nearly a year. His reason having returned, he claimed his liberty, and contrary to the advice of Esquirol and Marc, he obtained it. I considered him to be in a fair condition, but I had remarked, that when the linen was being prepared for the laundress his eye partook a peculiar expression as soon as he perceived the things which the women had used during the menstrual period. Some years afterwards he suddenly attacked his wife who lived with him, taking her for a demon, who reproached him with his crimes; she only escaped death by throwing herself out of the window. At the end of twelve days, C. expired in a hospital in the capital, furiously maniacal, believing himself surrounded by phantoms and devils.

C——, a melancholic monomaniac, subject to hallucination, was placed in my establishment under a delusion which led him to see enemies in every one he met, the consequences of which had been most deplorable. Possessed with this idea, he had resolved to attack any one who should persecute him with greater animosity than others. A poor man whom he did not know, was the victim of his illusions. One day, having seen a man enter a wine shop, who appeared, as he thought, to mock him in a very insolent manner, he seized a gun and fired it, killing the man on the spot. In consequence of this murder, he was put under my care. He was at first sombre and uncommunicative, but being calmed by isolation, and perhaps also, as has been frequently observed in similar cases, the result of his act having soothed his cerebral excitement, he replied better to the questions which I put to him. The following are the explanations which he gave to me:—"At first, and for a long time, I strove against my destiny; nothing succeeded with me; I finished by believing what was desired of me, that I had enemies; I heard voices threatening and reproaching me; I met numbers of persons who made grimaces at me and mocked me. It is probable that under the influence of these ideas, I have killed this man. I am very sorry for this misfortune; but I feel that my visions no longer exist, and that I am cured." A month after, he was set at liberty!

Five years elapsed, when he was brought back to me by order; one of his brothers, a prey to the same hallucinations and delirious conceptions, had assassinated a half-imbecile woman, and, as a measure of precaution, my old patient was sequestrated afresh. The conversations I had with him satisfied me that his hallucinations had ceased. He even reasoned tolerably well, but his ideas had no longer the same clearness; he could not give account of all that was demanded of him; his memory was weakened, so that he acquitted himself but indifferently in his business of a compositor. He told me that he had more than once found himself without means. He stayed at the house about three months. During his residence a third brother came several times to see him, who in his turn declared to me that he was melancholic, and said to me at different times, "I fear misfortune."

Some time after the departure of the brother who was confined by order, the one who had killed the woman was brought to me. He was a tall man, apathetic, uncertain in his looks, and presented all the symptoms of melancholic monomania. After the commission of the murder, he was a prey to terrible excitement, he roared like a wild beast, and was obliged to be confined in a strong room. When he was placed under my care, he was calm, but remained in his chamber, or walked about alone without speaking to any one. My inquiries apprised me that he was one of those withered branches, of which there are many, who because he had a smattering of learning, and made little verses, thought he should soon make a figure in the world. Discontented with his want of success, he conceived a hatred to society, and his melancholic character assisting, he entered within the fatal circle of enemies, threatening voices, mocking figures, &c. His brother observed to me that he was less mistrusted than himself, because he was less energetic. He was, however, watched, but he had concealed an old surgeon's knife, with which he did the fatal act. This second murder was also a consequence of the suggestions of the voices which incessantly harassed him, and made him believe that he was the butt to the persecutions of society. His victim was the scapegoat, who paid for all, when his delusion had transformed her into the inveterate enemy who embodied all the others. According to his own avowal, he knew little of this woman, with whom he had no connexion, but who resided in his neighbourhood. I questioned him at various times as to this sad event; he confined himself to saying that it was a misfortune, and appeared ill at ease, and I gathered from his expression, that to persist in the questions might lead to troublesome consequences. This patient had, like his brother, a certain slothfulness of idea, although he did not reason wrongly; if work was given to occupy him, he kept it for a considerable time, and he finished by doing nothing, saying that a sick man ought not to be fatigued. There could remain no doubt as to the changes brought about by mental alienation in his intellectual faculties; there was present a state of semi-torpor, indecision, and apathy, which is frequently observed in cases of this nature. He was no longer a madman, but a moral invalid, who was thenceforward to remain a charge to society.

On visiting, in 1846, Bethlehem Hospital, where are confined criminal lunatics, so called, Dr. Alexander Morrison showed us several patients, who in consequence of hallucination, and of these illusions of sight and hearing, had committed murder.

Making use of these cases, in which mental alienation was incontestable, to guide us in those analogous cases which not having been observed beforehand, or disclosing themselves suddenly, might embarrass physicians and lawyers, we are now about to relate several, of which two or three formed the subject of a medico-legal report made by ourselves.

Madame H——, large, strong, very well educated, but exceedingly romantic, and having always evinced exaltation, married at the age of twenty. For a long time her union appeared happy. As the critical time of life approached, her reason presented symptoms of very remarkable disorder. She imagined that her husband had sold her, and that he had caused her to be dishonoured before his own eyes. Her religious principles became extremely developed; she believed herself in communication with celestial intelligences, she heard divine voices; God made revelations to her. At this period, she began to feel a hatred to her husband which continually increased. She spoke incessantly of doing him acts of violence. Her sister frequently remonstrated with her on this subject. One day, when she was more pressing than usual, Madame H—— seized her by the throat, and attempted to strangle her, and throw her out of the window.

She was treated for this access in the establishment of Dr. Pressat, where she remained a month; when she was withdrawn she never spoke unreasonably, but she experienced great religious exaltation. She was continually in churches, where it appeared to her she saw marvellous things. On returning home, she was tolerably tranquil; her husband, however, who was uneasy, shut himself every night in his room. One night he heard a gentle knocking at the door, he rose immediately and asked who was there; no one answered. Half-an-hour later, some one knocked again; this time there was a response; it was madame, who said in a plaintive voice, "My dear, I am unwell; I have come to ask you to give me some assistance." The husband opened the door, and Madame H—— entered, and instantly struck him five well-aimed blows on the head with an iron bar. With a desperate effort he pushed her out, shut the door, and fell covered with blood.

Madame H—— was restored next day to the house where she had been treated the first time. At the end of some days, having become calm, she was unable to explain this act but by a derangement of her reason "I imagined," she said, "that my husband was metamorphosed into a devil, and I had a horror of him."

Several months after, this lady, who was then quite tranquil, was transferred to my establishment in the Rue Neuve Saint Généviève. Her conversation was reasonable and witty, but she preserved the same antipathy with regard to her husband.

Her sister, who was on the point of being her first victim, often came to see her; she loved her much, and expected her visits with impatience. When I asked her one day about the insensate attack she had made upon her, the lady answered me, "What would you have? When I threw myself upon my sister it appeared to me that her figure was that of a hideous green corpse, that her look was that of a devil; this spectacle caused me such horror that I wished to get rid of it at any cost." Similar reasons had caused her to strike her husband.

During her stay with me, this lady, who passed her days at work in my family, and in the evening made one of our party, was several times subject to these hallucinations and illusions. Notwithstanding the kind of life she lived, the reasonable nature of her conversation, her features had at times so sinister an expression that I had forbidden all persons other than the attendants to go to her apartment. When she was tormented by her illusions she uttered threats of death, against which we took precautions by shutting her up in her own room.

We cannot dwell too much upon examples of this nature, for they may give rise to the most unfounded commentaries, and to interpretations the most contrary to truth, so deceitful sometimes are the appearances! Such a fact happened with regard to an event which was reported in these terms in the *Bulletin des Tribunaux*:—

"We said in our number of 9th July, 1843, that an attempted assassination, surrounded with strange circumstances, had taken place in the *Place du Palais-Royal*. A young working jeweller, named Garnier, crossing this place about nine o'clock in the evening, found himself only a few yards from the post of the Chateau d'Eau, occupied by the Municipal Guard, when he heard the report of a gun. Garnier believed in the first instance that the shot had been directed at an officer who was walking near him; but he had scarcely spoken a word to this officer before he himself fell and lost his senses. Although he had only felt in the first instance a smart shock, a ball had nevertheless struck him and wounded him deeply in the abdomen.

"The author of this crime in the first instance escaped all the investigations of the police. Garnier, whose wound happily was not mortal, declared that he had had no quarrel, and that he knew of no enemies. Three weeks passed without the slightest discovery having been made, and the event appeared inexplicable, when a fortuitous circumstance at last put the police on the track of the presumed culprits. Warrants were at once issued against them, and the day before yesterday a commissary and several agents of police proceeded to the arrest of the persons implicated, named Raphael C. de G——, aged 27, born at Palma, residing at Paris, Rue St. Thomas-du-Louvre, No. 15, and Otto Fischer, Prussian domestic servant, Rue du Jour, No. 8.

"G—— opposed the commissary and agents with desperate resistance. His fury was such that although he had not had time to take up his arms, it required four of the strongest men to hold him, and who only accomplished it by securely binding his arms and his legs. They found at his house several loaded pistols, sword-sticks, three knife-poniards, balls, powder, &c.

"When the fury of this man was a little calmed, he declared that he was the sole author of the crime, and that it was wrong to have arrested Otto Fischer. He pretended that he had been grossly insulted by Garnier, and that he wished to avenge himself, but everything leads to the belief that G—— has an extreme interest in disguising the truth, and that the shot which struck the unfortunate Garnier was destined for another person. The examination is proceeding."

Would it not appear natural to conclude after reading this article, that M. de G—— was a great criminal who had the prospect of a seat on the bench of the Court of Assizes? Let us examine how the case ended. Scarcely had he undergone one interrogation before doubts arose in the magistrate's mind as to the integrity of his intellectual faculties. Dr. Brun, conjointly with another colleague, were charged to make a report upon the state of his reason. The conclusions were such that an *ordonnance de non-lieu* placed him at the disposal of the administrative authority, who sent him to Bicêtre.

The sensation which this hospital produced on a man born of a noble class, and having a comfortable fortune, was so great, that he made several attempts to kill himself by hunger.

After a short stay he was transferred to my establishment. The first impression was altogether in his favour; possessed of a good address and figure, an agreeable smile, black hair, expressive eyes like most Spaniards, and speaking with much politeness, he could not fail to interest those who saw him. I kept him quiet for a few days, and then I asked him the details of the events which had happened to him.

"As you see me, sir," said he, "I am the most unfortunate of men. For some years a vast conspiracy has been organized against me in my own country; the whole town of Palma is bent upon my ruin; relatives, friends, inhabitants, have united together to cause my destruction; they revile me, lay snares for me, make grimaces at me &c.

"In order to escape this persecution, I took refuge in France; I claimed the protection of the Prefect of Police; but I soon found that this change of place was useless, and that my enemies had suborned others, whom they gained over by money. For several days they did not leave me an instant's repose. Furious and impatient of this conduct, I fired on one of them who had heaped insults upon me, and who did not cease to make grimaces at me."

"Did you know the man, then?" I asked.—"I had never seen him before!"—"Permit me to observe that your answer appears very extraordinary."—"That's it; they wish to make me out a fool. But I declare that I am the judge of my own honour; whenever I am insulted, either I will kill my adversary or he shall kill me."

Some time afterwards he desired to speak to me in private.

"Sir," said he, "I perceive that my enemies are strong; I am ready to make great pecuniary sacrifices in order to get away from here. Tell me what sum of money will satisfy the government." I remarked that it was not customary in France to make people pay for their liberty, and that in all probability he would be sent home. Three months passed thus; at last one of his friends, sent from Spain by his family, having arrived, I gave M. de G—— into his care, recommending that he should not be left for an instant until he arrived at Palma, as he retained the same ideas, and an accident was always to be feared.

What a subject for reflection does this case present! With the exception of the fixed idea of enemies who insulted him, made grimaces at him, and sought to injure him, although he had never seen them, M. de G—— was like all the world. He conversed in an interesting manner about his own country and literature, he painted and sang very well. Again, he avoided allusion to the events which had occasioned his captivity. And, nevertheless, this man, who employed himself all day, would have killed the first person he might have met whom his delirium had transformed into an enemy.

The illusions of sight may exist alone, and they may be associated with those of hearing. They who suffer thus believe themselves to be victims of the most odious machinations.

Sad and melancholy ideas and fear concur singularly in impressing this direction on the mind. The fear of the police and of enemies has in a great measure taken the place of the fear of the devil and of spirits, although for some years demonomania has reappeared above the horizon. Nothing is more common than to be consulted on behalf of lunatics who are the butt of persecution, whom some one desires to poison or assassinate. I was one day called in to a lady who appeared to be in full enjoyment of her reason; she said to me with the greatest coolness in the world, "Sir, eight days ago, when I was going to mass, I perceived that I was followed by men of suspicious demeanour; on leaving the church I found three of them in ambush in the Rue de l'Ouest; one of them tried to spring upon me. The day before yesterday, the porter of my house placed a ladder against the wall in order to ascend to my room; he ran away on seeing me. On all sides people seek to do me evil. I am surrounded with assassins." In almost all cases this variety of monomania exists with hallucinations of hearing and of sight.

The patients who are tormented by these ideas imagine that people murmur at their ears disagreeable and offensive words, and that they are insulted. To escape these vexations, some seek isolation, frequently change their domicile, and make every effort to destroy their traces; others, of a more daring character, march up to their pretended enemies and provoke them to duel, and there is no doubt that unfortunate men have fallen under the sword of such madmen.

As the moral disorder increases, all the means employed by these patients to escape the snares of their enemies are without effect. The latter introduce themselves into their dwellings and harass them at every instant, address them in ironical, menacing, and insulting words, appear to them in the streets, in the silence of night, and the sufferers end by seeing an enemy in every figure they meet with.

When the disease arrives at this stage, the exasperation of the patient is sometimes such that he resolves to escape this frightful suffering by suicide. We have already quoted such cases. At other times, lunatics, furious at these persecutions, determine to avenge themselves; they strike, wound, or kill the first individual they meet with, who, according to their expression, pays for the others. Under some circumstances, they conceive a hatred for the person with whom they hold the most intimate relations, or whom they see most frequently, and their actions in such cases are liable to impose on superficial minds who look upon these proceedings from revenge.

Lunatics labouring under hallucinations of this class are in general very formidable. And examples are not wanting to justify this opinion.

M. R. de G——, a clerk in government employ, resided before he came to Paris in a provincial town, where his habits of life made him the subject of observation. He changed his hotel suddenly, taking his meals out of the house, no one knew where. At times he cooked during the night, and when he dined in town he would not touch the dishes until he had seen them tasted by other guests. His mistrust was such, that he fastened his door with several locks, and kept his visitors waiting a long time. In order to lead astray the curious, he spoke of projected journeys, which he had no intention of taking. His sombre and even unpolite character had raised enmities, which his superior sought to calm by making some well-meant representations: he replied coldly, that there was a society of poisoners directed by one Mérope (an imaginary person), whose agents followed him wherever he went, and had partially succeeded, as he experienced horrible pain in his bowels.

A short time after his arrival in Paris, he related to the clerks in his office, that he had seen a man hidden behind a hedge, who attempted to shoot him, or who at any rate took aim at him; but when he approached the murderer disappeared. He added, that he knew for certain that an individual, whom he was not able to distinguish, had come during the night to saw the bars of his chamber. In order to defend himself against these attacks he desired a clerk to lend him two pistols. On his way to Saint Germain by railway one day, he perceived in the carriage several persons, who eyed him with a menacing air: he left them, took a car, and next day purchased two pistols. Afterwards, according to his account, an unknown person sought to stab him with a poniard.

This patient saw nothing but malevolent enemies, who laid snares for him, spread calumnies, sought to hurt him and poison him. Every one pointed the finger at him, treating him as a fool, on account of his fears and his mode of life. He accused especially one, the superior in his office, of having done him a great injury in revealing his sufferings, which he had confided to him under the seal of secrecy.

Six years before, being at Fontainebleau, he had overheard two Englishmen reading a mysterious letter, in which there was no mention either of him or of any one of his acquaintance, but the terms in which it was conceived, and the conversation of the strangers, led him to think that these were persons suborned to assassinate him.

This *halluciné*, who was always armed, declared that he had several times been on the point of using his weapons, but that he had waited to fire until the individuals should have advanced and touched him.

Under the influence of this idea, M. R. de G—— waited upon M. D——, chief of the staff in the office, and under excitement which he admitted, he fired two pistol-shots upon this superior officer, who had become in his eyes the personification of his pretended enemies, and attempted to commit suicide.

No physician hearing this series of events would fail to recognise a monomaniac subject to hallucination. What is important to remark is the idea of poisoning, the continual apparition of malevolent personages, which during more than eight years had not prevented M. R. de G—— from fulfilling with distinction the administrative career which he had embraced, and on the eveof his arrest he corrected a report which does not indicate the slightest mental derangement.

The Chamber of the Council of First Instance of Paris, after a long examination and upon a medico-legal report, made by M. Foville and ourselves, dismissed the charge against M. de G——, remitting him to the disposal of the Prefect of Police.

During the month of May, the Commissary of Police was called upon to take cognizance of a murder. The accused appeared deeply sorry for his crime, he declared before the public officer, that he struck M. M—— because everybody wanted him and urged him to do it, and mocked him, but that he was not animated by any motive of hatred, he had simply desired to revenge himself on some one. The evidence of witnesses showed that after having worked industriously for seventeen years in a warehouse, he had left under pretext that people there murmured offensive proposals in his ear, and that he was made a butt of; since then he believed himself to be sought after by the police.

On being asked to explain why he had attacked M. M—— with an instrument of iron freshly sharpened, he replied, "I was followed by evil-disposed persons; one had seized me by the throat in the Faubourg St. Denis. Some months previously I became aware that I was followed in the dark by five or six individuals walking behind me, who said, *He must be killed—he must be killed.* I scarcely ever set foot in the street but some one whispered in my ears disagreeable and insulting proposals; they called me an assassin and a thief. I therefore sharpened the end of a foil, saying to myself, since people are disposed to kill me, I must defend myself."

Transferred to Bicêtre, after the report which we made in concert with the Inspector-General Ferrus, Soyez there passed several months in a state of apathy. On one of our visits we learnt that he had struck one of the overseers with a knife without any provocation; the latter related, as follows, how this came to pass:—"Two months ago, Soyez approached me in a cheerful manner; he had scarcely made two or three steps when, after having looked at himself in a mirror, he came rapidly upon me and struck me with a knife on the right side, so violently that the blade was broken on my key and some pieces of money which I fortunately had in my pocket. While striking, and afterwards, he reproached me with burning him, and attempting to burn his wife and child. My opinion is, that he is subject to hallucinations."

On being interrogated, Soyez acknowledged that he had formerly had delirium, but that he was cured. When spoken to about the overseer, he admitted also that he had wandered for a moment; he added:—"He burned me, and I reproached him with it; besides, he is always burning me." He afterwards conversed with the physicians of persons who jumped upon his body, and of extraordinary things which he saw during the night.

No doubt could remain as to Soyez's mental condition, and that under the influence of his fixed idea, and of his hallucinations, he had twice committed such serious acts: the physicians also considered that during these two occasions he was not in possession of his free will; that his actual mental condition, and the danger there would be in setting him at liberty without being certain of his cure, made it necessary to keep him in custody. These conclusions were adopted by the public minister. We have since learned that the mental disease of Soyez has increased and that it is considered incurable.

Lunatics under hallucination, who are convinced that they have enemies, and hear their voices, their insults, their threats—who believe that people make grimaces at them—that the faces of those who surround them express hatred and a desire to injure them, or else that they assume the features of monsters and devils, all common phenomena in melancholic monomania, ought to be the object of rigorous watchfulness, for science teaches that these cases are subject to frequent relapses.

A countryman had had a maternal uncle who became insane; he was married, and for several years lived on good terms with his wife, when it was perceived that he became sombre, mistrustful, irritable, and often evinced a fear that some one wished to kill him; hallucinations and illusions served to intensify his delirious conceptions. It was remembered on the inquest that he named one Robert as the instigator of the plots laid against him. On the 3rd of May, 1828, D—— went to bed after having kissed his wife, and without giving any indication of the double crime he was about to commit (his wife being pregnant). The next day, 4th of May, D——'s wife was found to have been killed in her bed with a mallet. An examination took place, and the counsel for the prosecution came to the conclusion that D—— should be declared to be in a state of dementia. The tribunal was not of this opinion, and ordered a supplementary examination; D—— was sent to Paris in the month of September in the same year, and was placed in the lunatic ward at Bicêtre, in order to be under the observation of Esquirol and Ferrus.

After a residence there of several months he became more communicative, and on the 14th of April, 1829, a marked change was observed in him; he appeared more disturbed and troubled.*

* This change in expression, words, and habits which a lunatic undergoes, is of extreme importance. Every time we have noticed it, the individual has had a crisis, and has made attempts at murder, suicide, escape, &c.

It was noticed that he had hallucinations and illusions of hearing. On the 18th, D—— went to bed without the attendants remarking that he was more excited than on previous days. During the night, feigning a desire to ease himself, he left the dormitory, and got a broom-handle, with which he beat a patient who slept in the sixth bed from his own. On being seized by the attendants, he allowed himself to be confined in a strait-waistcoat, *affirming that he heard voices* which told him to avenge himself, and he added, that they were quite right to restrain him, for he intended to do the same to two or three others.

On the report of Esquirol and Ferrus, the tribunal dismissed the accusation, but nevertheless ordered that D—— should be placed at the disposal of the proper officer in order that he might take the necessary measures regarding him to ensure the public safety and his own interest.

This observation recals that of Pinel's patient, who, after fifteen years at Bicêtre, assassinated one of his companions under the influence of illusions, as he had already killed, under similar circumstances, another individual before he entered the hospital.

If we have present to our minds the symptoms we have described, if we remember that the chief delirious conception entertained by the category of lunatics comprised under the designation of melancholic monomaniacs (Lypemaniacs), is a belief in the existence of enemies who to them are often embodied in some real or imaginary person, as in the preceding observations, towards whom they bear insatiable hatred and whose death they determine upon, it will be found exceedingly likely that many great political assassinations may have been committed by madmen under the influence of hallucinations. It is well known in the present day, that lunatics compass plans of evasion, that they prepare secretly the means of striking their victims, of avenging themselves on those by whom they consider themselves offended, and that in the concoction and perpetration of these plans they exhibit cunning, address, discernment, and intention. The impassibility which many of these individuals have shown under punishment belongs to the unsound condition of their mind, to that tenacity and obstinacy of idea which nothing can overcome, and to a physical phenomenon common amongst them, consisting of an extreme insensibility of the skin and tissues known under the name of anæsthesia, which is sometimes so extreme that they are seen continually to tear off their skin and burn and mutilate themselves.

M. Bazin, in his *Histoire de la Fronde*, relates that Ravaillac, during his examination, replied that some days before his crime there exhaled from his feet stifling smells of sulphur and fire, which demonstrated to him the purgatory which heretics deserved.

Another time, he felt a body jump upon his person. Several days before the assassination he had seen sacramental wafers rise in the air and place themselves beside him. Finally, he added, that in a certain town he had seen a head of a Moor on the body of a statue, and that having desired a painter to give it him, he found the head again at the painter's, from which he concluded that Henri IV. was as black as a devil; that he could never wash away his sins, and that he was damned for ever.

Historical documents show that we must reckon Jacques Clement as a lunatic subject to hallucinations.

"One night, as he was in bed, God sent his angel to him, who presented himself to the monk in a great light, and showing him a naked sword, said these words,—'Brother Jacques, I am the messenger of the Almighty, and am come to certify that by thee the tyrant of France must be put to death. Think of it, and prepare thyself as the martyr's crown is already prepared for thee.' This said, the vision disappeared and left him to dream of these remarkable words. The morning come, Brother Jacques recals before his eyes the preceding apparition, and doubting what he ought to do, addressing himself to an old friend, also a monk (Father Bourgoing, prior of his monastery), a scientific man, well versed in the Holy Scriptures, to whom he frankly declares his vision, asking him at random if it is displeasing to God to kill a king, who has neither faith nor religion."

The young German who attempted to strike Napoleon at Schœnbrunn also had visions: he saw the Genius of Germany, who bade him deliver his country.

MacNaughten, the assassin of Drummond, was persuaded that he was surrounded by malevolent people who threatened him, and he saw everywhere strange figures.

The French General de S——, who was stabbed a few years ago by one of his relations, was also the victim of one of these madmen under the influence of hallucinations.

It is painful to think how many persons have fallen by the hand of similar lunatics, and we believe that many such deplorable events might be avoided by not waiting until these dangerous madmen should have realized their threats.

The impulses and actions attributable to hallucinations and illusions are not observed merely in acute delirium, mania, and melancholic monomania: they are met with also in various other forms of madness. Thus, amongst monomaniacs we find dangerous orders given. Puerperal madness, on account of the predominance of the melancholic type, often presents a propensity to suicide; it existed in several of our cases. Out of 111 instances of this kind of delirium noted at Bethlehem, and published by Dr. Webster, thirty-two were remarked as having a tendency to suicide. Authors have quoted observations of infanticide amongst women suffering from puerperal madness. The motives to these murders were hallucinations of sight and hearing. We have several times had occasion to advise the removal of children from their mothers because their lives were in danger. In stupidity a great number of actions, apparently automatic or without relation to external objects, have subsequently been explained by the influence of hallucinatory phenomena; this is a new argument in favour of the opinion that the most eccentric actions observed in monomaniacs and especially in maniacs, are always caused by hallucination or illusions.

The degradation of the intellectual faculties, the state of dementia even, are not obstacles to the production of hallucinations and illusions, because it is not demonstrated that under either of these circumstances no part of the brain remains healthy. The same observation holds with regard to general paralysis. Five of our patients believed themselves surrounded by enemies, thieves, and assassins, and called to the keeper with all their force, and sometimes with frightful shrieks. These illusions induced a paralytic to spring out upon his servant in order to strangle him. Another broke all the glass in his window with a view of jumping into the court to escape malefactors. M. B——, a paralytic lunatic of four years' standing, fancies he sees from time to time a shark ready to devour him. He then utters cries which are heard a long way off, beats the walls of his room, and one day attempted to kill his sister with a razor.

This hallucination was attended with very serious consequences. One of his cousins, who was present at this scene, was so alarmed that she experienced a suppression on the spot, to which she succumbed a few days after. I have thought it necessary to recal, as briefly as possible, a certain number of cases quoted in the body of the work, because they clear up some obscure points of legal medicine.

The discussion which arose at the Academy out of M. Trousseau's communication on the case of epilepsy which was taken for cerebral congestion, has furnished several remarks of great importance from a medico-legal point of view. Thus, M. Tardieu has established that the epileptic seizure which generally attacks all the senses, may, in certain cases, only affect the will. M. Devergie has affirmed that it is never during the attack, but in the interval between the attacks, that criminal thoughts date their birth, and always in cases of confirmed epilepsy.* M. Trousseau has proclaimed that if a man suddenly commits a murder without any previous intellectual disorder, without having,

* "Discussions sur les congestions apoplectiformes;" *Gazette des Hôpitaux*, 7 Mars, 1861.

up to that time, evinced any signs of madness, without being poisoned by alcohol or other substance capable of exercising an energetic action on the nervous system, and irrespective of any passionate action, that man is almost certainly an epileptic. Further on he adds :—"*I say almost certainly*, if I have not seen the attack ; but if I have seen the grand access or the initial vertigo immediately preceding the incriminated act, I then affirm absolutely that the accused has been incited to the crime by a force which he has been unable to overcome."* Finally, M. Baillarger† has set himself to prove that besides actual madness there exists in certain epileptics a special intellectual and moral condition.

But amongst all the questions treated by these eminent authors, there is one which has been completely thrown aside—it is that of the relations between hallucination and epilepsy in what M. Tardieu has so well named the epileptic shock (choc). So long ago, however, as the first edition of my *Traité sur les Hallucinations*, in 1845, I called attention to this subject, reported observations in support of my views, and wrote this sentence—"Sometimes the fantastic figures address the epileptic in words, they insult him or command him to do something. It is probable that many crimes committed by these unfortunate beings, and of which some have been severely punished, were but the result of hallucinations of hearing and of sight"‡

We have insisted on this subject in this third edition, and have pointed out the frequency of hallucination with epilepsy.

These hallucinations are generally of an alarming and sinister nature. Several of our patients were dazzled by a strong red light, which shone like lightning before the access. One of them in the instant before the loss of consciousness saw a diabolical figure approach him like the shadows of a magic lantern; he cried aloud, Here comes the devil! and then fell to the ground. Dr. Gregory's patient saw before the access an old woman in a red cloak, with a wicked expression and hideous features, who struck him on the head with a stick. He had scarcely received the blow when he fell down in convulsions, perfectly unconscious.§ A countryman told us that in an access which preceded his admission to my house, whilst working in the field he seized a scythe and commenced cutting everything before him, incited by a voice which bid him do so. After having traversed a great

---

* Trousseau, "Des déterminations subites et irrésistibles dans leurs rapports avec l'epilepsie," *Union Médicale*, 19 Mars, 1861. Caspar, *Traité pratique de Médicine Legale*, 1861, 2 vols. in 8vo.

† "Note sur la Responsabilité des épileptiques," *même journal* 21 Mars.

‡ A. Brierre de Boismont, 1re ed. p. 193 ; 2 ed. p. 208, 21 suiv.

§ Paterson.

extent of arable land, he stopped, worn out by fatigue, beneath a wall, and there fell asleep. If he had happened to meet with any living creatures what misfortunes might there not have been to deplore? Esquirol, who had remarked the extreme terror caused to epileptics by their hallucinations, asked if it might not be this feeling which impressed on their physiognomy the character of fright or indignation which belongs to these patients during the access.

Hallucination and illusion ought then to be taken into consideration with reference to the instinctive and sudden acts of epileptics.

The determinations, the actions to which lunatics are impelled by hallucinations and illusions may be the consequence of dreams during sleep, or the intermediate state between sleeping and waking, or may take place during somnambulism. Under some circumstances no delirium has preceded the outbreak.

On the 1st January, 1843, a young man presented himself at an inn near Lyons, and sought supper and lodgings for the night. About ten o'clock at night the landlord heard a great noise in the stranger's room. He hastened upstairs, but had scarcely entered when he was struck with the blade of a pair of tailor's scissors. The young man having been seized and disarmed, was asked the motive which induced him to make the assault. He replied that he had *seen* the innkeeper kill two men, that he had *heard* him plot his own assassination, and that he had then determined to sell his life dearly. Having been transferred to the prison of Lyons, the accused in all the interrogatories to which he was submitted, evinced good sense and ordinary intelligence. He related afresh all that he had *seen, heard,* and *felt.* His narrative was that of a man convinced of what he stated, devoid of passion, and with the air of one who rejoiced at his escape from a serious danger. On the report of Drs. Chapeau and Tavernier, who were charged to inquire into his mental condition, and after the examination conducted by the *juge d'instruction,* this individual was set at liberty.

These facts, which are more common than is generally supposed, and which depend on the impression produced by dreams, an impression sometimes so strong that it persists through life, deserve to be remembered and well considered. One can scarcely refrain from shuddering with regard to this case, at the frightful position of the accused if he should have killed the innkeeper, and at the difficulty of his justification if he had chanced to have any motive of ill-will against his victim, if it had been but a dispute about the price of his repast, or if there had been any ground to impute an intention to theft.*

---

* *Bulletin des Tribunaux,* 20 Jan., 1843.

Illusions of dreams continuing up to the moment of waking, and even in a state of wakefulness, may give rise to singular, reprehensible, and even criminal acts. We have several times been witness of extraordinary scenes which were but a continuation of dreams. The individual spoke and acted under their influence; one might have been tempted to take him for a madman; but presently the images of the night weakened and disappeared, and he was one of the first to be astonished at his language and his actions, although he asserted that at the moment his sensations appeared to him quite natural. Under this impression, men of great presence of mind when surprised by ordinary danger, have evinced dismay, which could only be explained by the state out of which they were passing.

"A quartermaster of one of the regiments of the *Chasseurs d'Afrique* took up his quarters for the night at an inn where the *salle à manger* was decorated with a paperhanging representing the most glorious of our feats of arms on the African territory. In the middle of the night the innkeeper heard an alarming uproar in the *salle à manger*; it turned out to be the quartermaster in his shirt, who, having got out of bed under the influence of hallucination, and with a billet in his hand, was engaged in mutilating the Arabs on the walls. There was great difficulty in making him understand his error, and he was let off on payment of the damage."*

In the following case the result was most deplorable.

"Bernard Schidmaizig started out of his sleep in consequence of a frightful dream. At the moment he perceived near him a horrible phantom. Terrified by the obscurity, he imagined that the apparition approached him, and arming himself with a hatchet, he struck the spectre, and killed his wife."†

The hallucinations of natural somnambulism have also some importance in legal medicine. The curious history which Brillat-Savarin has preserved in his *Physiologie du Gout*, and which we have reported in our work, proves incontestably that nocturnal hallucination may be the cause of crime.

The Neapolitan journals have instanced the case of a man who, dreaming in a fit of somnambulism that his wife, who slept in the same bed with him, was unfaithful, wounded her dangerously with a dagger that he always carried about him.‡

To these two examples we add the following: "The father of Lord Culpepper, so well known by his dreams, appeared to take his trial at the Old Bailey, in 1686, for having killed a guards-

---

* *Journal de Belfort*, 24 Aug. 1843.

† Macnish, *The Philosophy of Sleep*, 3 ed. p. 87.

‡ *Union Médicale*, 16 Dec. 1851.

man and his horse. He pleaded somnambulism, and was acquitted; having produced about fifty witnesses who testified to extraordinary things done by him during sleep."*

Marc, therefore, has good ground for saying that the state of sleep deserves special attention in the medico-legal examination of madness. In fact, with the majority of maniacs it is troubled, and broken by visions, hallucinations, terrors, panics, groanings, and vociferations. Too much care cannot be given to the watching of those melancholic monomaniacs whose sleeplessness is almost continual. Silence, shadows, and obscurity redouble their terrors, leaving a clear field for the working of their hallucinations; it is also during the night or early morning that they put in execution their sinister projects. With many monomaniacs, even those whose dominant ideas are cheerful, sleep is equally broken and difficult, because in the absence of repose, and in the excitement of the day, their imagination abandons itself with greater facility to conceptions whence spring delirium.

The superficial observer finds it difficult to trace the source of a great number of actions apparently incomprehensible. To the moralist this circle is already greatly limited; but to the eyes of a physician especially, the thick veil behind which so many men believe themselves hidden, becomes, so to speak, transparent: and he finds in their temperament, their defects, their passions, their vices, their maladies moral and physical, the natural explanation of their conduct. Thus, for example, to confine ourselves to the subject in hand, hallucinations and illusions better understood in our day, account for numbers of acts deemed inexplicable or attributed to depravity, bad dispositions, or crime.

Amongst the facts of this nature, we have directed special attention to a variety of melancholic monomania, complicated with hallucinations. We have proved by numerous conclusive observations, the evidence of which has satisfied magistrates (since we have no theory on the subject), that many individuals who pass for quarrelsome, hot-headed, provoking persons, and even murderers, belong to this category.

The question of confinement touches too closely on our subject to permit of its being passed over.

It is evident that when hallucinations are inoffensive, and do not disturb the operation of the mind in the ordinary affairs of life, recourse should not be had to sequestration: it may, indeed, have the serious inconvenience of rendering the patient entirely insane. In the case of the English naval officer who perfectly performed the duties of his profession, but believed that he could see Napoleon in the sun, Dr. Conolly observed, with justice, that

* Macnish, p. 195.

this vision having no influence on the actions of the officer, his confinement in an asylum would be reprehensible.

We entirely agree with Dr. Conolly; and we think with him that individuals ought not to be sequestered because they hold peculiar or even eccentric ideas on special subjects, otherwise a new field would be opened to arbitrary power. A man may be looked upon as singular or mad because he believes in the existence of two worlds—the one visible, and the other invisible; that there is no real solitude; that every out-of-the-way place is inhabited by spirits; that there is no action, howsoever secret, that has not numerous witnesses. Nevertheless, in avowing this belief, he says nothing that has not been taught by religion; but if he go a step beyond, if he pretend to communicate with these invisible beings, he runs the risk of being considered a madman, although many great men have believed in the reality of these things, and of late years many reasonable persons and some well-instructed physicians have imagined that they have communicated with rapping spirits: he is, in fact, under the influence of an hallucination: he has allowed his thought to take to itself a body: his power of comparison and his judgment have failed him. But even though he should deceive himself on this point, if his conduct is becoming, if his actions are blameless, no one has a right to intervene in his affairs, or to call him to account for his opinions, much less to place him under confinement. We will go further: if the hallucination exhibits itself in a man deeply religious, if it relates to his convictions, if it is not contrary to common sense, we see nothing in this belief which should cause him to be considered as a madman.

Thus, whenever hallucination is inoffensive, isolation is not necessary; but it is otherwise when it may be prejudicial to the individual or to others: sequestration is then indispensable. Cases of mutilation, suicide, homicide, theft, arson, and calumnious denunciation, are so common amongst persons labouring under hallucination, that there is no occasion to insist upon this point. The same precaution ought to be taken with regard to monomaniacs who are subject to the hallucination that they are surrounded by enemies, as soon as they commence to threaten, because experience has too well taught us with what instantaneousness they are led to commit acts of violence.

Does responsibility attach to the actions of persons under hallucination? At first thought the affirmative would not appear doubtful. But how can we think it right that an individual should be prosecuted, who may have killed some one because a voice, to him real and all-powerful over his mind, has ordered him to do it; or because the victim appeared to him to wear the garb of a violent enemy, a murderer ready to immolate him, a frightful monster, a demon, &c.? The question is more embarrassing when the accused appears to have been moved by revenge, and to have acted with discernment and premeditation: such was the case of Luigi Buranelli, who was hanged in London in the year 1855, for having killed one Lambert, with whom he lodged, and who had turned him out because he had had improper relations with a woman who also lodged with him. It appeared, however, from the facts that the character of this man had suddenly changed since the death of his wife: that he had become melancholy, irritable, and inclined to suicide; this melancholic disposition had suggested to him the idea of enemies who sought to kill him, and whom he personified in two individuals of his acquaintance. Besides this, he was under an illusion of sight which caused him to see his bed inundated with water.*

The *Journal de Médecine et de Chirurgie* related some years ago the condemnation of a foreigner subject to hallucination; the hullucination was incontestable, and the court and jury based their conclusion on the fact, that it had not had a direct influence on the crime, and that the accused was conscious of his act.

It is true that there are persons under hallucination who distinguish their false sensations and resist them; but such cases are rare. It suffices to have lived in asylums, public or private, to satisfy one's self that there are others who will tell you: I know well that these voices are false, and that my cries are absurd, but I cannot free myself from my hallucinations, nor refrain from crying out — there is something within me stronger than my will.

Interdiction may be had recourse to in cases of hallucination, when the nature of the delirium is such that it may involve the ruin of the individual and that of his family. The patient, who at the instigation of a voice threw all his fortune down a well, might have been saved from misery if this measure had been applied in time.†

The paralytic, whose case we related in the memoir on the prodromic period of general paralysis, would not have ruined his family, and obliged them to disperse themselves in different parts of the world; and would not himself have died a pauper in a public asylum, if interdiction had been pronounced in good time. Thirty

---

* Forbes Winslow, "The Case of Luigi Buranelli Medico-Legally Considered."—*Journal of Psychological Medicine*, vol. viii. 1855.

† Does any one desire another conclusive example of this power of voices? Here is one which was related to me yesterday by M. Am. Latour: "A man of my acquaintance had lived 72 years in the practice of the duties imposed by the Catholic religion. All of a sudden he heard a voice which said to him, *That which thou hast hitherto believed is false, absurd!* The metamorphosis was complete; his religious sentiments were extinguished, and he is now as incredulous as he was believing! Had the voice said to him, *Kill*, the incredulous had become a murderer!"

years of practice have proved to us how often this measure is indispensable; but while acknowledging its necessity in well defined circumstances, we do not forget that nearly ten years ago we wrote as follows:—Privation of civil rights ought not to be decreed on account of an original kind of life, peculiar conduct, eccentric speeches, or the belief in imaginary facts, which in no way compromise the fortune of the individual, or expose him to be the dupe of intriguers.*

In support of this opinion, we quoted a judgment of the Court of Appeal of Paris which rejected a demand for interdiction made against a Demoiselle D——.

It was, however, impossible not to see in this case, which we have reported at length in our work on the interdiction of the insane, one of the best established examples of hallucination and illusion. The detailed examination of Dr. T—— and the opinion of medical experts, left no doubt on this point; but though the existence of hallucinatory phenomena was incontestable, it was no less true that Mademoiselle D—— had been in nowise influenced by them in her conduct, that her acts were in no way blameworthy, and that her answers to interrogatories did not indicate insanity; we, therefore, entirely coincided in the conclusions of the court.

Testamentary capacity is no less connected with our subject than are the questions of sequestration, responsibility, and interdiction. There are degrees in everything—the absolute is impossible; we must analyse cases and see if a method applicable under one set of circumstances might not be hurtful under another. There is no need to demonstrate that one cannot accept as valid the will of an hallucinated testator to the disinherison of his family, who falsely considers his relations to be his enemies without cause of complaint, or imagines that they wish to poison him, and have mingled noxious substances with his food, employed electricity to torment him, or subjected him to infectious odours.

In the work already quoted on interdiction, which appeared some time ago, we pointed out the serious consequences which this state of mind might lead to in the case of the insane.†

Liberty of mind is not admissible when the person under hallucination takes the figures of his imagination for those of devils or monsters, &c., or when he imagines them to utter words of menace or insult. In these cases, as in those where the hallucinations and illusions have a direct and baneful influence on

---

* *Des Hallucinations*, p. 707, 2 ed. 1852.

† Brierre de Boismont, "De l'interdiction des aliénés et de l'état de la jurisprudence en matière de testament dans l'imputation de demence, avec des notes de M. Isambert, conseiller à la cour de cassation."—*Annales d'Hygiène et de Médecine Légale*, 1852, t. xlvii. p. 108. Paris, 1852.

the actions, the wills of the patients cannot be legally sanctioned, for the reason that the volition no longer exercises itself reasonably.

The conditions are altogether altered when the testator, in spite of his hallucinations and illusions, is conscious of what he does, and directs his conduct according to the ordinary rules of human wisdom, and shows, by the composition of the testamentary act, that he enjoyed his faculties, that from the commencement to the termination he kept the same end in view, and, in a word, that the false sensations have exercised no influence on his conduct. The testamentary capacity is then intact and the testament cannot but be maintained.

*Resumé.*—Hallucination from the profound conviction which the patient entertains of its reality may be the cause of a great number of hurtful, reprehensible, dangerous, and criminal determinations.

The frequent recurrence of threats, insults, morbid interpretation of words, transformation of figures and objects in acute delirium and mania, lead to consequences which are often very painful.

The unhappy sensations of melancholic monomania, often more decided than in the preceding forms, and which are more especially characterized by the sight of persons making grimaces, of enemies, and by the hearing of menacing words, culminate in numerous attempts by the patient against himself and others.

Suicide, so frequent in this form of madness, is determined by menaces, reproaches, and terrifying visions.

Melancholic monomaniacs who believe themselves to be the object of plots and persecutions, are excessively dangerous. A considerable number of murders are committed by persons of this description. Provocations to duel have often been the consequence of mental disorders of this nature.

Sometimes suicide is the result of a sudden hallucination or illusion.

Hallucinations due to ideas of ruin, persecution, enemies, poisoning, accusation of theft, condemnation, damnation, &c. frequently lead to suicide.

Voices from invisible sources are often the causes of culpable acts.

In cases of this nature it is necessary to avail one's self of an acquaintance with all the antecedent circumstances; and, in case of doubt, to have recourse to isolation, which at times will be required for a prolonged period.

In many cases patients yield to a superior force.

The hallucinations and illusions to which drunkards are subject, and which have been called "ebrious," have over and over again occasioned suicide, murder, rape, and arson.

The actions to which individuals are drawn by hallucinations are sometimes suddenly evolved and determined on. Night, shadows, and solitude appear to favour this disposition.

Illusions, as well as hallucinations, may be the determining causes of theft, arson, mutilation, assassination, &c.

Illusions of sight and hearing have a considerable, and often irresistible, influence on the conduct of the insane.

It is probable that political assassinations have been committed by lunatics, subjects of hallucinations.

Hallucinations and illusions are a key to numbers of actions apparently incomprehensible.

The impulses and actions due to hallucinations and illusions are observed also in monomania, puerperal madness, dementia, and general paralysis.

The hallucinations which precede the epileptic shock form an important element in the actions of these madmen.

The hallucinations of sleep and of the transition from sleeping to waking, and *vice versâ*, and of natural somnambulism, should be taken into consideration with reference to the perpetration of acts committed by lunatics.

M. Baillarger remarks with great reason, that those hallucinations which precede sleep, often lasting from the very first for several hours, are a cause of transitory madness, and will frequently account for actions committed during the night by persons who on the morrow are perfectly sane. Isolation is often necessary in hallucinations, but sometimes it is to be avoided. The exception should not be allowed when the ruin of the individual or of his family is at stake; but it should be granted in the case of a person who is inoffensive, and where the hallucinations do not pervert the judgment.

Hallucinations are no obstacle to making a will, when they have not existed a long time; if they have exercised no influence on the general conduct; if they have not weakened the natural affections, and if the person under their influence has always satisfactorily performed his social duties.

---

